# Barriers and Advances in Kidney Preservation

**DOI:** 10.1155/2018/9206257

**Published:** 2018-12-04

**Authors:** C. Steichen, S. Giraud, D. Bon, B. Barrou, L. Badet, E. Salamé, T. Kerforne, G. Allain, J. Roumy, C. Jayle, P. Hannaert, T. Hauet, R. Thuillier

**Affiliations:** ^1^Inserm U1082, Poitiers, F-86000, France; ^2^Université de Poitiers, Faculté de Médecine et de Pharmacie, Poitiers, F-86000, France; ^3^CHU de Poitiers, Service de Biochimie, Poitiers, F-86000, France; ^4^CHU de Poitiers, Département d'Anesthésie-Réanimation, Poitiers, F-86000, France; ^5^CHU de Poitiers, Service de Chirurgie Cardio-thoracique, Poitiers, F-86000, France; ^6^Fédération Hospitalo-Universitaire SUPORT, Poitiers, F-86000, France; ^7^IBiSA Plateforme ‘Plate-Forme MOdélisation Préclinique, Innovation Chirurgicale et Technologique (MOPICT)', Domaine Expérimental du Magneraud, Surgères, F-17700, France

## Abstract

Despite the fact that a significant fraction of kidney graft dysfunctions observed after transplantation is due to ischemia-reperfusion injuries, there is still no clear consensus regarding optimal kidney preservation strategy. This stems directly from the fact that as of yet, the mechanisms underlying ischemia-reperfusion injury are poorly defined, and the role of each preservation parameter is not clearly outlined. In the meantime, as donor demography changes, organ quality is decreasing which directly increases the rate of poor outcome. This situation has an impact on clinical guidelines and impedes their possible harmonization in the transplant community, which has to move towards changing organ preservation paradigms: new concepts must emerge and the definition of a new range of adapted preservation method is of paramount importance. This review presents existing barriers in transplantation (e.g., temperature adjustment and adequate protocol, interest for oxygen addition during preservation, and clear procedure for organ perfusion during machine preservation), discusses the development of novel strategies to overcome them, and exposes the importance of identifying reliable biomarkers to monitor graft quality and predict short and long-term outcomes. Finally, perspectives in therapeutic strategies will also be presented, such as those based on stem cells and their derivatives and innovative models on which they would need to be properly tested.

## 1. Introduction

Kidney transplantation remains the treatment of choice for many patients with end stage renal disease and is a superior long-term therapy compared to dialysis in terms of quality of life and life expectancy. During the transplantation process and particularly the preservation step, a certain degree of ischemia-reperfusion injury (IRI) inevitably occurs in the immediate posttransplant setting. Ischemia-reperfusion (IR) process plays a significant role in the pathogenesis of both delayed graft function (DGF) in allografts and hemodynamic mediated acute kidney injury (AKI) of native kidneys. [1]. This clinical problem is exacerbated by the current situation, which is characterized by a shortage of organs driving to the use of marginal donors. Indeed, despite the extracorporeal cold preservation protocol used worldwide to overcome this issue, graft injuries related to IR are frequently observed and caused by pathophysiological mechanisms directly related to nonoptimal preservation strategies.

The main issue is that there is no clear consensus regarding optimal conservation solution composition, oxygenation, hypo- or normothermic conservation, and perfusion method [2], stemming directly from the fact that as of yet, the mechanisms underlying IRI are not entirely defined and the role of each of these parameter not clearly outlined. In the meantime, as donor demography changes, organ quality is decreasing which directly increases the rate of poor outcome. This situation has an impact on clinical guidelines and protocols and impedes their possible harmonization in the transplant community, which has to move towards changing organ preservation paradigms: new concepts must emerge and the definition of a new range of adapted preservation method is of paramount importance.

This review presents existing barriers in transplantation (e.g., temperature adjustment and adequate protocol, interest for oxygen addition during preservation, and clear procedure for organ perfusion during machine preservation), discusses the development of novel strategies to overcome them, and exposes the importance of identifying reliable biomarkers to monitor graft quality and anticipate short and long-term outcomes. Finally, perspectives in therapeutic strategies will also be presented, such as those based on stem cells and their derivatives and innovative models on which they would need to be properly tested.

## 2. Kidney Preservation: Where Are We Starting from?

Organ preservation contributes to the induction of injuries induced by decreased ATP production, acidosis, cellular edema, and mitochondria alterations [3, 4]. The choice of preservation solutions is thus critical. Experimental models showed that (i) the ionic composition needs to be close the plasma's potassium (K^+^) concentration (≈5mM) and sodium concentration (Na^+^) (≈140 mM), in order to limit hyperpotassic effects (membrane depolarization, vasoconstriction, and consequently low perfusion) [5], and that (ii) the presence of molecules exerting an osmotic and/or oncotic pressure to prevent edema is essential to optimize graft quality [6]. Many solutions of different compositions are commercialized, such as University of Wisconsin solution (UW), Custodiol (HTK), Celsior, and fourth generation solutions such as Solution de Conservation des Organes et des Tissus (SCOT-15) and Institut Georges Lopez-1 (IGL-1), all with various colloids and ionic composition. A strong corpus of experimental results in preclinical models is available for SCOT-15 and IGL-1. These solutions include polyethylene glycols as colloids, a suitable option to protect organ integrity/functionality [5–9], and their characteristics are presented in Table 1 (adapted from Bon D et al. [1]).

Ten years ago, a multicenter analysis of kidney preservation drew several conclusions: (1) kidneys from deceased donors should ideally be transplanted within 18 hours; (2) within the 18-hour window, the time of ischemia has no significant influence on graft survival and (3) UW solution should be used if longer preservation is envisioned [10]. HLA matching improves graft survival regardless of length of ischemia [10]. This meta-analysis included 5 different conservation solutions and 91,674 patients, mostly brain dead donors. Unfortunately, these observations are not adapted to the current donor demographics which includes a growing number of suboptimal donors, such as Extended Criteria Donors (ECD) and Donation after Circulatory Death donors (DCD). Indeed more recently, a clinical study studying brain death donors (including ECD, 3939 patients) showed that each additional hour of cold ischemia time beyond 6 h significantly increased the risk of graft failure and mortality [11]. In addition, keeping the cold ischemia time as short as possible has also been shown to be crucial during machine perfusion [12]. However, the true impact of ischemia time is still debated with diverging conclusions, especially for donors displaying AKI [13, 14]. A need for wisely triaged donors is absolutely mandatory.

Organs from DCD donors or ECD are more susceptible to preservation injury and have a higher risk of unfavorable outcomes, and there is thus growing need for new potential and standardized protocols for organ preservation. Concepts such as machine perfusion (MP), temperature, and other technical advances need to be assessed through rigorous common networks and research programs, with a complete characterization and rationalization of solution composition, preservation temperature, the exact role of oxygen, and the most adapted perfusion protocol.

## 3. Adding Oxygen during Preservation: Is It Time to Take a Breath?

One of the hallmarks of current organ preservation methods is hypoxia/anoxia. Indeed, unpublished data from our laboratory show that the oxygen dissolved in the preservation solution is consumed within the first two hours of kidney preservation.

In the absence of oxygen, mitochondria are able to maintain some protonic gradient and produce ATP as long as supplies last, accumulating succinate [15]. However, when oxygen is reintroduced in the system at the reperfusion stage, it is captation of a single electron which produces superoxide anion, the first reactive oxygen species (ROS), and source of oxidative stress. If not controlled, the production of ROS and subsequent destruction of structures is fatal to the cell. ROS production is also mainly responsible for the destruction of the glycocalyx at the cell surface and consequence lesions, among which coagulation and sterile inflammation [16].

Oxygen thus appears to be a two-edged sword which should be wielded carefully. However, experimental evidence tends to show a majorly beneficial use of oxygenation. In the current context of unavoidable donor pools expansion, oxygen supplementation during hypothermic preservation is the focus of numerous preclinical and clinical studies, including nonheart-beating, heart-beating, and higher-risk donors [17–19]. Naturally called for, the use of oxygen in normothermic preservation is considered elsewhere [2, 18, 20].

Several methods have been used in animal models to provide oxygen during storage: oxygenated perfusate or perflurocarbon emulsion, hyperbaric oxygenation by the delivery of oxygen under increased atmospheric pressure, or retrograde persufflation of gaseous oxygen bubbled through the renal vasculature [21]. Several studies have investigated hyperbaric chambers as a mean to oxygen delivery and demonstrated that perfusion was necessary to improve function, rather than static storage, hinting towards the need for active delivery within the organ rather than changing the outside conditions [22, 23]. In a pig kidney model, retrograde oxygenation also showed beneficial compared to static storage [24]. However, both techniques of hyperbaric preservation or retrograde persufflations are difficult to envision within a clinical setting. Therefore, efforts have been deployed to use perfusion in order to deliver oxygen. Interestingly, oxygenation pressure was again shown to alter outcome, with the benefits being lost at higher pressure (60/40mmHg) [25, 26]. These studies however did not compare oxygenated perfusion to standard perfusion.

In canine, comparison of standard machine perfusion with the Lifeport to oxygenated perfusion on the RM3 did not demonstrate a difference in outcome, albeit with a short follow up and with light IR lesions (45 min warm ischemia) [27]. This study is an example of the animal models limits: study animals are healthy, and machine perfusion is already a good preservation method; therefore measuring the impact of optimization may be difficult without a necessary increase in the level of IR lesion (either through lengthened warm ischemia or marginal donor modeling). Superiority of oxygen addition was also demonstrated when using an oxygen emulsion in machine perfusion, also in terms of function recovery in canine [28].

Addition of oxygen to the perfusion circuit was tested. Our own group studied the use of oxygen in a machine intentionally designed to deliver it: the Kidney Assist. In a pig model of deceased after circulatory death donor (60 min warm ischemia), we demonstrated that oxygenation improved early function recovery as well as long-term outcome (in terms of function and fibrosis development) [19]. When compared to historical data using either the Lifeport or the RM3 in a similar model, we showed that oxygenation indeed permitted better early recovery, but long-term outcome was within comparable range (Unpublished data).

It thus appears clear that oxygenation is a very promising avenue of optimization for organ preservation, particularly if coupled with machine perfusion. Numerous mechanisms are involved in the benefits of active oxygenation at 4-8°C, mostly the ability to produce some ATP and maintain cellular and repair processes [29]. However, in both static and dynamic hypothermic preservation protocols, actual levels of oxygen within the kidney remain largely undetermined, as well as oxygen consumption. Unpublished data from our laboratory assessed oxygen and ATP in static and machine-preserved kidneys (20 hours; n = 5* per* group) using our established preclinical porcine model of severe warm ischemic injury (WI, 1 h), mimicking donation after circulatory death. WI reduced tissue ATP by 90% (control: 2.6 ± 0.5 mM). In both preservation protocols, PO_2_ decreased rapidly (t_1/2_~1h) from atmospheric levels to 51.8 ± 0.2 mmHg and 7.6 ± 0.2 mmHg, respectively. During machine perfusion, arterio-venous (av) oxygen consumption was calculated (QO_2_, *μ*mol/min* per* kidney) and was 3.5 ± 0.1* versus* 1.6 ± 0.6 *μ*mol/min* per* kidney in static preservation. Post-preservation, tissue ATP amounted to 5.4 ± 0.6 and 0.1 ± 0.01 mmol/L in machine and static, respectively. Despite profuse assertions and hypotheses in the field, this is the first comparison and quantification of renal oxygen levels, oxygen consumption and associated ATP levels in standard, non-oxygenated static and machine preservation. This type of study of effective renal graft oxygen levels (and consumption) should translate into a better understanding of the graft's requirements and open the way to improvements of organ preservation devices and conditions.

## 4. Preservation Temperature: Should We Really Keep It Cool? 

The drive to use hypothermia for organ preservation naturally stems from fact that, on a biochemical point of view, lowering temperature slows cells metabolism, through two relations:

(i) The van't Hoff equation: At 4°C, average temperature of hypothermic organ storage in transplantation, a chemical reaction will only be 40% as effective as the same reaction taking place at 37°C.

(ii) The Arrhenius relation, quantifying the impact of temperature on the speed of a chemical reaction, highlighting that a reaction taking place at 4°C is 90% slower than the same reaction at 37°C.

However, as organs are not test tubes in which run single chemical reactions, but complex structures deploying a plethora of reaction and interactions, the validity of hypothermia may be put into question. Indeed, molecular structures, such as hydrogen and hydrophobic bonds, are deeply affected by hypothermia. Thus, both proteins and lipids structure and therefore function are altered when lowering temperature. For instance, it been demonstrated that the ability of transcription factors to bind DNA is affected by temperature [30].

In this context, hypothermia conservation is being questioned by the scientific community, and numerous articles highlighted that hypothermia indeed worsens ischemic injuries through; (i) reduction of ATP synthesis and metabolic activity [31], (ii) reduced Na-K-ATPase activity, which induces osmotic perturbation [32], (iii) mitochondrial perturbations, (iv) decreased cell survival [33] and (v) endothelial activation [34, 35]. Optimization of organ preservation temperature is thus a pivotal goal [36].

Among emerging concepts of alternative storage temperatures, recent studies advocate the use of normothermia (35-38°C), subnormothermia (25-34°C) [36–38] or mild-hypothermia (12-24°C). The use of normothermia may be considered for the whole preservation or combined with periods of hypothermia [36] and aims to restore normal cellular processes while facilitating viability assessment or to prepare organs to reperfusion. Data from our laboratory focusing on endothelial cells submitted to different temperatures* in vitro* during hypoxia show that subnormothermic temperatures provided protection against injuries* versus* 4°C, by reducing cell death, mitochondrial dysfunction, leukocyte adhesion and inflammation. However,* ex vivo* pig kidney evaluation on a perfusion apparatus showed that the benefits of 19°C or 32°C were limited, with similar levels of tissue preservation damages (submitted manuscript). This study suggests that temperature optimization for kidney preservation will require thorough investigation, combining the use of complementary relevant models and the design of elaborated preservation solution and new technologies.

Additional data from our laboratory studying the impact of temperature on the cytoskeleton showed using* in vitro* model of renal endothelial cells submitted to cold ischemia (4°C) that, while intermediary filaments were unaffected, cells microfilaments showed radical changes with disappearance of the structure replaced by a disorganized array of nodules; moreover, microtubules almost completely disappeared with time [39]. Furthermore, temperature, and not oxygen deprivation or the solution, was the determining factor of the cytoskeleton's loss of integrity during preservation.

Obviously, the specifications for normothermic preservation may require an oxygenated perfusate with an oxygen carrier or blood itself and use of MP. In addition, perfusate for normothermic perfusion will mandate elaborate compositions including nutrients, anti-oxidant and metabolic substrates. Subnormothermic dynamic preservation aims to avoid cold-induced injury without increasing metabolism to a level at which intense oxygenation requires an oxygen carrier. These elements will be further discussed below since most of subnormothermia or nomothermia protocols are performed on dynamic preservation.

## 5. Kidney Perfusion: Pump It up!

Preservation time with cold storage (CS) is limited as prolonged CS increases the risk of delayed graft function (DGF) that contributes to chronic complications. Furthermore, the growing demand for the use of marginal donor organs requires methods for organ assessment and repair. Machine perfusion has resurfaced and dominates current research on organ preservation. Since 2009, compared to cold storage, MP benefits are demonstrated in terms of reduced risk of DGF, risk of graft failure, and improved graft survival [37, 38]. However, the donor populations in which MP should be applied have not yet been resolved but it seems that there is no reason to limit MP to marginal kidneys. Indeed, extracted from UNOS database from 2005 to 2011, a review showed that, similarly to marginal kidneys, MP is beneficial in reducing DGF even when standard donors are considered [40]. Our laboratory identified the benefits of kidney MP as being mediated by endothelium releases of the vasodilator nitric oxide (NO), due to shear stress activating the endothelial NO synthase (eNOS) by phosphorylation, resulting in improvement of cortical microcirculation (measured by laser Doppler) [41].

Another non negligible advantage of using MP during conservation is the possibility to assess organ quality. Organ resistance during MP has been described as predictive value for graft survival (initial resistance) and DGF (resistance measured after 2 hours of MP) [42], although this is still debated. A more valuable approach is offered by the machine giving access to the organ perfusate throughout the preservation period, allowing the measurement of biomarkers predictive of transplantation outcome. Indeed, an international study showed that GST, NAG and H-FABP were independent predictors of DGF but not of primary nonfunction and graft survival [43]. In addition, rapid metabolomic analysis in the perfusate by nuclear magnetic resonance showed, in a preclinical model, that the levels of several metabolites during MP are associated with function recovery [44].

Regarding temperature, MP is optimal to test alternative temperatures such as normothermia or subnormothermia. Hypothermic dynamic preservation aims to slow down cellular metabolism and counteract undesirable and detrimental effects of ischemia. It combines low temperature (4–10°C) with an acellular colloid-containing preservation solution using, in the majority of cases, the Na-gluconate/hydroxyethyl starch MP solution developed by Belzer et al. [45]. Subnormothermic machine perfusion at temperatures of 20 – 25°C potentially allows elimination of cold-induced injury without increasing metabolism too high, since normothermic preservation mandates an oxygenated perfusate with an oxygen carrier (usually red blood cells) [18] complicating the process. A pilot study demonstrated the superiority of Lifor Preservation Medium (a complex organ preservation medium containing sugars, amino acids, buffers, colloids, fatty acids, antioxidants, vitamins, dextran and an oxygen carrier) at room temperature perfusion compared to Belzer machine perfusion both at room temperature and 4°C, in a porcine model of uncontrolled donation after circulatory death [46]. In an acellular normothermic perfusion system, the use of Oxygent (a complex fluid supplemented with an oxygen carrying perfluorocarbon emulsion) was able to preserve canine kidney autografts using pulsatile preservation at 32°C and static storage at 25°C [28]. Such data underline the evidence for a technological evolution of cold storage concepts.

A published economic evaluation, using a Markov model with a 10-year time horizon, showed that life-years and quality-adjusted life-year can be gained while reducing costs at the same time, when kidneys are preserved by MP instead of CS [47]. However, several questions regarding the optimal machine perfusion system still remain unanswered. Future research needs to explore optimal perfusion modalities such as oxygen used and concentration, pressure, pulsatility, temperature. In addition, optimal perfusion solution (enriched perfusion medium, whole blood leucocyte-free blood etc.) need to be carefully investigated (machine vs. solution effects) [48]. Finally, the question of timing is of utmost importance [37]; at present it is unknown whether brief hypo- sub- or normothermic MP following CS is sufficient to renal reconditioning or if CS should be completely replaced by MP.

## 6. Donor - Organ – Recipient Conditioning

Machine Perfusion associated or not wit extracorporeal circulation procedures could also be used as a tool to condition the donor before organ procurement. Abdominal regional in-situ perfusion (ARP) has been applied clinically at hypothermic and normothermic temperatures in organ donors. These methods have been found to improve kidney graft function, to replenish ATP and to reduce injury in a number of large animal models [38]. The first alternative is called In Situ Cooling and consists in performing organ cooling by using diluted blood solution previously cooled at 0 to 20°C. The second option called NRP for Normothermic Regional Perfusion, consists in using the donor blood to perfuse the abdominal organs before collection. NRP is the preferred form of donor management in uncontrolled / unexpected donation such as DCD donors [49, 50], compared to* in situ* cold perfusion and total body cooling [51]. Reports from different groups in Europe, the USA, and Asia have described the use of NRP in both uncontrolled DCD and controlled DCD kidney transplantation, with rates of delayed graft function approximating 50% and 30–40%, respectively; negligible (if any) primary no function; and excellent one-year graft survival [25, 52–55].

Perfusion could also be used to condition the organ itself, such as at the end of a static preservation phase, in order to “wake up” the organ before its transplantation. Several reports reported that abrupt change in temperature from hypothermic preservation to normothermic reperfusion at the time of transplantation produces detrimental effects on renal graft quality [18, 37]. Recently, Controlled Oxygenated Rewarming (COR) of grafts immediately before transplantation has been described as a modification of MP, bringing a new approach for organ conditioning and strengthening the concept of a pretransplantation organ preservation and evaluation unit. COR following CS demonstrated superior results over MP for liver and kidney [20, 56], avoiding “heat shock” and possibly the side effects (including mitochondrial dysfunction) of quick rewarming [57]. Subsequent studies showed that COR improves renal function after reperfusion (better renal creatinine clearance) and protect mitonchondria integrity [58, 59].

Another alternative technique is to recondition a kidney preserved by hypothermic preservation (either CS or MP) using 2 h of normothermic perfusion with blood. This short period of* ex vivo* normothermic perfusion (EVNP) immediately before transplantation, has a positive conditioning effect on the graft [60]. A first clinical case published in 2015, demonstrates the feasibility and safety of this technique [61]. A proof of concept clinical trial is currently being carried out in the UK to validate this technique. Alternatively, a slow and controlled increase in temperature up to normothermia using a combination of acellular medium and autologous erythrocytes addition is also currently under evaluation [56, 62].

Finally, at the end of the chain, recipient conditioning could also be applied: recent reports suggested potential therapeutics to protect organs from reperfusion injury, such as remote ischemic conditioning [63]. Other approaches are also interesting with the use of molecules such as statin (HMG-CoA reductase inhibitors).

## 7. Additives in Preservation Solution: Improve the Now While We Wait for the New

Improving preservation may not necessarily require revisiting the composition of the solution or its temperature, as indeed several compounds have shown the ability to significantly improve quality when added to existing technology. We previously published an extensive review of molecules which could thus be used [1], and are proposing an update below. Some targets such as mitochondria integrity and/or permability, innate immunity, anoxia and O2 transport, endothelial cell integrity and coagulation pathways are outlined in this section [64].

Firstly, several studies have shown that coagulation pathway was one of the key to counteract IRI. Coagulation inhibition takes multiple forms, uncovering the complexity of this pathway. As a first example, in a mouse model of liver IRI, the Protease activated receptor (PAR)-4 pathway was targeted [65], while a clinical study showed that PAR-1 is expressed by DCs in DGF grafts and its activation may induce complement production and a Th1 bias [66]. Secondly, in a mouse model of hepatic IRI, recombinant human thrombomoduling was protective, and specifically this activity was brought through the N-terminal lectin-like domain 1 (D1) subunit, involving TLR4 signaling [67]. Moreover, anticoagulants have demonstrated efficacy, such as an anti-Xa molecule protecting against preservation injury in a pig autotransplantation model [68]; a novel multi-arm heparin PEG conjugate adsorbing the endothelium and protecting against hypoxia* in vitro* [69]; a mast cell heparin proteoglycan mimetic (APAC), which was shown to be more effective than heparin in protecting against renal IRI in rats [70]; and finally a dual anti-Xa/IIa compound which was successful in limiting reperfusion injury in a pig kidney autotransplantation model [71].

The involvement of complement in IRI has been extensively demonstrated in a variety of mouse models [72] and prompted the testing of complement-targeted therapies against IRI [73] and the initiation of clinical trials to test the benefits of an anti-C5 antibody (Eculizumab) to prevent DGF (Delayed Graft Function) (NCT01403389; NCT01919346), which are still ongoing. Eculizumab treatment in pediatric kidney transplantation permitted better early graft function and improved graft morphology, however there was an unacceptably high number of early graft losses [74]. Inhibition of C1 protease using a recombinant human inhibitor (RhC1INH) inhibited complement deposition in a large animal model of kidney warm ischemia [75] and reduced fibrosis in a mouse model of warm IR [76]. Moreover, this treatment was able to protect kidney grafts, when used only during the reperfusion phase, against acute and chronic IRI in a pig model [77]. Finally, this inhibitor was used in a Phase I/II clinical trial to measure the impact on need for hemodialysis during the first week post-transplant, with significant reductions in need for dialysis and improvements in long-term allograft function observed with C1INH treatment [78]. C3 also appear a viable target, either in vascularized composite allograft model with a targeted inhibitor [79] or at the donor level, when the inhibitor was given as a nebulized solution priori to lung transplantation in a mouse model [80]. Moreover, targeting the alternative pathway also appears beneficial, through for instance the administration of anti-factorB antibody in a mouse kidney transplantation model [81]. Finally, a novel membrane-localized complement inhibitor based on a recombinant fragment of soluble CR1 (APT070, Mirococept) is currently tested in patients (EMPIRIKAL trial, REC 12/LO/1334), offering the possibility to treat the donated kidney before transplantation [82].

Additionally, a natural oxygen carrier extracted from Arenicola marina with high oxygen affinity developed as an additive to standard organ preservation solutions showed a protective effect in a variety of experimental conditions [83, 84]. A novel non-steroidal mineralocorticoid receptor antagonist was recently studied in kidney IRI models and its protective effect was well established [85, 86].

Enhanced understanding of cell and mitochondrial behavior during preservation is paramount to improve outcome. Several promising avenues of research are emerging from the study of hibernating species [87], such as the use of H_2_S [88]. Other concepts include: the replacement of damaged mitochondria with healthy mitochondria at the onset of reperfusion by auto-transplantation in the heart [89]; the control of pH regulation through inhibition of carbonic anhydrase in lung transplantation, which impacts both pCO_2_ levels, and Na-K-ATPase expression [90]; the sensitization of calcium channels in human hepatocytes for liver transplantation [91]; or the control of systemic iron load to protect against renal ischemia-reperfusion injury-associated sterile inflammation [92]. Finally, other drugs were recently studied: pharmacologic targeting of DHPS by N1-guanyl-1,7-diaminoheptane (GC7) or RNA interference–mediated inhibition of DHPS or DOHH induced tolerance to anoxia in immortalized mouse renal proximal cells [93].

## 8. Studying Organ Preservation: Where Are the Top Models?

The quest for innovative strategies relies on the availability of predictable models recapitulating as physiologically as possible transplantation-induced IR injury. The models currently available are of 3 types:* in vitro* cultures of renal cells,* ex vivo* perfusion of isolated renal structures/organs and* in vivo* models of kidney ischemia reperfusion and/or transplantation.

*In vitro* models include culture of renal primary cells or renal cell lines. Both of these cellular systems are cheap, flexible and compatible with high-throughput screening. Indeed, primary tubular cells for instance are able to temporarily keep the architecture, function and polarity of renal epithelial cells [94]. However their proliferation is limited and a de-differentiation rapidly occurs in culture [95]. This is why immortalized cell lines are widely used but their physiological relevance is questioned based on the modification they harbor to proliferate extensively.

In classical cell culture conditions, the Petri dish is composed of one major cell type with cells spread in two dimensions (2D), whereas the adult kidney is a complex organ composed of 26 cell types and displaying highly complex cell-cell and cell-environment interactions. Thus, experiments performed on* ex vivo* isolated kidneys are of high interest to predict the organ answer to complex stimuli. Rodents or pig kidneys can be collected and perfused on complex apparatus with buffered solutions or whole blood, in an attempt to maintain “normal” physiological/biochemical conditions in a closely monitored perfusion system. In our laboratory and others, pig kidneys are often chosen for their high similarity with human's and placed in a home-made perfusion apparatus in hypothermic or normothermic conditions, mimicking organ conservation or its reperfusion during transplantation into the recipient. Of note, perfusion systems are disconnected from extrinsic regulatory control mechanisms allowing targeted evaluation of the kidney, real time assessments of various parameters reflecting its state and function, as well as its response to different situations without confounding systemic responses that are present in* in vivo* studies [96]. The main limitation of these systems are (i) the lifespan of the organ (a few hours with regular system, however using specific perfusion systems 24 hours of conservation may be possible [97]), (ii) the necessity to have access to animal kidneys.

Finally, only* in vivo* animal experiments allow long-term follow-up of the organ function, and therefore represent the most predictive model especially when performed in large animals. In our laboratory, we have developed a pig preclinical model of kidney auto-transplantation which is invaluable to analyze various mechanisms and treatments in relation to ischemia reperfusion during transplantation [68, 93, 98, 99].

However, mainly for obvious ethical reasons, it is of crucial importance to avoid or limit the use of animals for experimentation whenever possible. In this light, cutting edge technologies can be applied to the field of IR research. Indeed, since 2006 and the first publication describing induced pluripotent stem cell (iPSCs) technology, it is possible to cultivate in the laboratory human iPSCs able to differentiate into all cell types of the human body. Recently, this technology has been combined with 3 dimensions (3D) culture systems to differentiate those cells into complex structures, highly resembling a tiny organ, called organoids [100]. They can be obtained in a 2 step protocol: firstly, cells are differentiated in a 2D monolayer using growth factors and cytokines which mimic first steps of* in situ* kidney embryonic development. During differentiation, cells are detached and placed on a suspension culture system enabling the further differentiation/maturation and auto-assembly of the cells in 3D. This protocol lead to the formation of spherical structures of a few millimeters in diameter (after ≈25 days of differentiation) intricately organized, vascularized and presenting 8 types of renal cells; authors note the presence of nephron-like structures with evidences of cells from distal tubules, loop of Henlé, Bownman's capsule, parietal cells, podocytes, epithelium from the collecting duct connected to the nephrons as well as a stromal population and endothelial capillaries.

Thus, working* in vitro* with human kidney-like miniaturized structures becomes possible. Additionally, use of iPSCs allows choosing the kidney organoid's genotype. This is possible either by selecting patients affected with one disease of interest to generate iPSCs and further differentiate them into kidney organoids to study renal disease mechanism and treatment [101], or by using genome editing technologies (CRISPR/Cas9) [102] to target specific genes of interest and study how they impact kidney organoid's response to various stimuli including hypoxia/reoxygenation protocols and resistance/sensitivity to conservation.

## 9. Improving Kidney Transplantation Outcome: What Else?

Recent advances in regenerative medicine brought new potential strategies in the field of organ transplantation. Among them, cell therapy (i.e injection of cells, usually stem cells or their derivatives) to repair or replace tissues is at the forefront of personalized medicine. Controlling or reducing IR injuries with cell therapy is a tempting approach. Most cell types that have been tested in the context of renal IR are mesenchymal stem cells (MSCs) from various origin, despite some studies describing the use of endothelial progenitor cells (EPCs) [103, 104] or cells differentiated from pluripotent stem cells [99, 100]. Importantly, most MSC-based cell therapy approaches have been tested on rodent models of IR-induced AKI, but not on models involving kidney transplantation. Overall, these studies show that administration of stem cell therapy improve global renal function, decreasing fibrosis and tissue damage and augmenting animal survival [105–108].

Cell injection timing has been revealed important. One study highlighted that pre-treatment with MSCs (7 days before IR induction) is more efficient than post-treatment (1 day after IR induction) to reduce lesions, this being probably due to a protective effect triggered by lipid metabolism modulation [109]. In our laboratory, using a pig preclinical model of kidney auto-transplantation, we choose the inject MSCs 7 days after kidney transplantation and observed significant improvement of kidney structural integrity and function [98]. Yet, optimal cell injection timing is far from consensual and this issue will have to be carefully studied in relevant preclinical model. Indeed, cell administration route and dosage are two critical factors which may be crucial for cell therapy efficacy: a comparative study observed that 1 × 10^5^ MSCs injected through the renal artery produces a dramatic improvement in renal function and morphology in rat model of renal I/R injury [110].

However, regarding MSCs at least (since the issue can be different for iPS-derived cells for example), there is no strong evidence that the cells are indeed able to graft or even remain in the kidney after their injection, and their protective effects does not appear to rely on their ability to differentiate and replace damaged tissues, but are primarily mediated by paracrine mechanisms. Thus, most approaches under development focus on the use of cell's secretome, instead of cells themselves. This is possible either by the use of conditioned medium (medium that was placed in contact with the cells for a period of time allowing cell secretion of paracrine factors and cytokines) or microvesicules (MV) directly isolated from the conditioned medium. These are extracellular vesicules important for cell-cell communication and containing miRNAs, mRNAs and proteins. Among paracrine factors identified as important for repair after IR are VEGF [111], Ang-1, and Ang-2. [103] and Glial-derived neurotrophic factor (GDNF) [112]. In the case of acute renal IR injury, the literature shows that MSCs contribute to the recovery of mice with IRI-induced AKI primarily through the release of MV [113]. Another study shows that MV from adult rat renal tubular cells significantly improved renal function in rats through a large transcriptomic shift [114]. Of note, exosomes can be injected alone or in combination with MSCs [115], hence an appealing option would be to combine MSC-derived exosomes with cells that are indeed able to graft and differentiate into kidney tissue such as iPS-derived kidney progenitors.

Among MV components, miRNAs are also a potential therapeutic target* per se.* The role of miRNA in IR was uncovered through a mouse model with genetic deletion of Dicer, enzyme involved in miRNA maturation [116]. This deletion lowered miRNA expression by approximately 80% and was shown to be protective against kidney bilateral I/R. While this approach was highly unspecific, the demonstration was made that miRNA were involved in I/R injury development. The same study showed that IR profoundly affected the miRNome after 12 and 48 hours of reperfusion, with at least 14 targets demonstrating a more than 2 fold change. Another study on mice subjected to 30 min kidney IR confirmed miRNome dysregulation [117]. Other studies in small animals have confirmed the alteration of miR-21 after IR [118]. Interestingly, this target was shown to play an important role in Ischemic Preconditioning (IPC), an efficient technique to ameliorate damage by IRI in different organs like heart, brain, liver, and kidney in several animal models [119–121]. miR-21 has several pro-apoptotic targets, hence the hypothesis that its overexpression could protect against cell death during IR. Indeed, in a rat model it was demonstrated that IPC induced miR-21 expression and subsequently protected against kidney IR, an effect that was negated by treated IPC animals with anti-miR-21 [122].

Likewise, long noncoding RNAs (lncRNAs) constitute a new class of noncoding RNAs that interfere with gene expression and are also involved in the progression of I/R injury such as myocardial, cerebral, hepatic, renal and mesenteric I/R injury [123]. For example, hypoxia-induced long non-coding RNA Malat1 (Metastasis Associated Lung Adenocarcinoma Transcript 1) has been described to be upregulated in renal I/R injury [124].

Additionaly, preconditionning or pre-treatment of MSCs is also a valuable option: IL-17A-pretreated MSCs resulted in significantly lower acute tubular necrosis scores, serum creatinine and BUN of mice with IRI-AKI [125]. Additionally, hypoxia-treated MSCs attenuate AKI through enhanced angiogenic and antioxidative capacities [126], mimicking organ preconditioning. Thus, such approaches can be combined and renal IR in rats was modulated by combination of ischemic preconditioning and adipose-derived mesenchymal stem cells (ADMSCs) [127].

Finally, in recent years, gene therapy has been developing, both in terms of targeting and efficacy. In transplantation, several studies have shown the feasibility of such an approach to improve IRI. As an example, in the liver, hepatic stimulator substance (HSS), a protein demonstrated to improve mitochondrial function, was overexpressed (through adenoviral transfer) and conferred resistance to IRI. siRNA can also be used intravenously, for instance to silence the expression of TNF-*α*: in a lethal kidney ischemia model, this was effective in protecting against IRI [128]. Finally, the stability of siRNA can also permit it to be used during preservation, improving outcome [129].

All these strategies will have to be carefully tested for their safety and short and long-term efficacy in predictive and pertinent models, as we discussed in the last paragraph.

## 10. Predicting the Future: The Importance of Biomarkers

Detection of chronic allograft injury remains a challenge after kidney transplantation. The objective is to define non-invasive biomarkers, both for graft quality evaluation during machine perfusion and graft function in the recipient. Mixed advances have been made to search for biomarker at the earlier step of the transplantation process, during machine perfusion. A clinical metabolomic study of machine perfusion perfusates showed differences in the metabolomic profiles for kidneys with immediate graft function (IGF) and delayed graft function (DGF) [130].

At the recipient stage, transplantation success is determined with measures of biochemical parameters such as serum creatinine or histopathological biopsy analysis, an invasive method. But the best biomarker of early graft function remains undetermined. Most of the time, scoring systems lacks sensitivity and specificity to achieve unanimity at an international level. The efficiency of creatinine as the best measure of kidney state remains questionable, since its inexpensive and easily implemented measurement is biased by certain physiological parameters such as tubular secretion, the influence of muscular mass or protein intake* via* nutrition [131]. And most importantly, it is a late marker.

Urinary NGAL, KIM, L FABP-1, Cystatin C, and IL18 were proposed as tools for early detection of acute kidney injury (AKI) but the determination of their validities and clinical utility is still in progress [132]. Concerning short-term outcomes, the presence of urinary IL-18 and NGAL immediately after transplant was associated with increased risk of delayed graft function [133]. Long-term outcomes may also be predicted with associated risk of graft failure and death correlating with elevated urinary tubular injury biomarkers such as IL-18, NGAL, NAG, and KIM. Some of these markers also have particular physiological importance and their presence can be related to structures alterations in specific part of the nephrons such as in proximal tubule structure alteration (KIM1, IL18, and FABP-1) or distal tubule (NGAL, FABP) [134, 135]. Recently, the cytokine IL-33 has been identified on rats as an alarmin contributing to kidney IRI by promoting iNKT cell recruitment and cytokine production, resulting in neutrophil infiltration and activation at the injury site [136]. However, IL-33 potential as a biomarker for kidney transplantation outcome has not been properly tested yet.

Besides “classical” biomarkers, different investigation paths are followed; some of them are listed below: 

*(i) Chemokines*. Two multicentric studies highlighted chemokines as an early predictive tool for kidney rejection, CXCL9 and CXCL10 mRNA especially [137]. CXCL9 mRNA and protein levels showed a negative predictive power [138]. CXCL10, CD3*ε*, and 18S RNAs allowed the distinction between antibody-mediated and borderline rejection [139]. 

*(ii) Exosomal Urinary NGAL.* It has been suggested that NGAL in the exosomes fraction could be more specific to evaluate renal damage because exosomes should be a representation of the physiological sate of the organ while whole urinary NGAL is not only specific for kidney damages [140]. 

*(iii) Serum Uromodulin*. Lower level of this kidney-derived glycoprotein was associated with risk of kidney allograft failure [141]. 

*(iv) Epigenetics and miRNA Regulation*. Aberrant DNA methylation patterns are already used as biomarkers in cancer, but only a few studies evaluated their role in transplantation. In kidney transplant recipients with subclinical rejection, long‐term allograft outcome was better when FOXP3+Treg cells were present, a subtype characterized by unmethylated locus near the FOXP3 gene [142]. Similarly, the Klotho promoter is hypermethylated in renal tissue and in peripheral blood mononuclear cells of patients with CKD, with the degree of hypermethylation correlating with the clinical and histological severity of CKD [143]. Another promising tool is circulating and urinary miRNA: numerous miRNA associated with kidney disorders has been reported and some of them in the case of transplantation [144]. A panel of 22 urine miRNA measured 3 months after transplantation allowed prediction of chronic allograft dysfunction (CAD) [145]. 

*(v) Epithelial-to-Mesenchymal Transition (EMT) or Endothelial-to-Mesenchymal Transition (EndMT)*. Both these processes are of high interest since they generate fibrosis and are induced by several molecular signatures among them TGF beta, EGF, and FGF2. A noninvasive approach has been developed for predicting fibrosis* via* assessment of the mRNA expression levels of genes implicated in EMT fibrogenesis such as Vimentin and CD45 [146]. Retrospective evaluation of EMT markers (Fascin1, Vimentin, and Heat Shock Protein 47) by immunochemistry in biopsy samples showed that they are a sensitive and reliable diagnostic tool for detecting endothelial activation during antibody-mediated rejection and predicting late loss of allograft function [147].

While the relationship between recipient kidney injury biomarkers and outcomes is relatively clear, the relationship between donor kidney injury biomarkers and recipient outcomes is more complex [134, 148]. In order to lift the hurdles preventing the discovery of new early and effective biomarkers, the generation of biobanks [149] and the use of laboratory for reconditioning the organ [150] (machine perfusion, ex vivo circulation, for instance) are important perspectives to set up projects looking for “ideal” biomarker.

## 11. Conclusion

**Figure 1:**
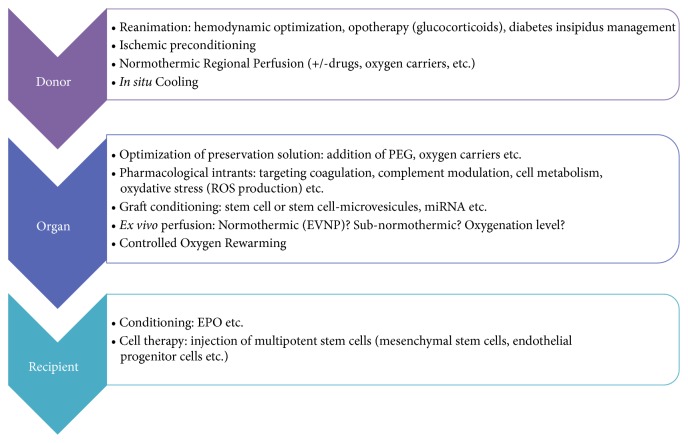
Strategies to overcome existing barriers in kidney preservation during transplantation.

While static cold storage is still widely used, and alternative means and solutions to optimize organ quality during its preservation exist in multiple and of various forms (Figure 1). The identification of the donor population which will most benefit from these strategies or combination of these strategies is also a critical question. The complexity also relies on the fact that all the preservation parameters (temperature, oxygen, static or perfusion, etc.) are dependent factors which need to be scientifically evaluated in independent experiments using models which all have their own limits.

There is an urgent need to promote translational research programs for the development of new clinical protocols. Members of the transplant community (academia, the biotechnology and pharmaceutical industries, and funding agencies) need to engage in an active dialogue and collective effort to find and advance therapies for organ preservation, but we need to assemble the evidences and target key questions in one unified effort.

## Figures and Tables

**Table 1:** Characteristics of current kidney preservation solutions and machine perfusion.

**Solutions**	**K** ^**+**^	**Na** ^**+**^	**Buffer**	**pH**	**Impermeant**	**Adenosine**	**Anti-oxidant**	**Colloid (g/L)**
	**(mM)**	**(mM)**				**(mM)**		
**Flush and Static cold storage**								

Blood	4.25	139	HCO3-	7.4	+	0	+	Albumine (50 g/L)

HTK (Custodiol®)	10	15	Histidine	7.2	+	5	-	-

UW (Viaspan®) (Bridge to life®)	100	28.5	(K)H2PO4 HEPES	7.4	+	5	Glutathion	HES (50 g/L)

Celsior®	15	100	HEPES	7.3	+	0	Glutathion	-

IGL-1®	30	125	(K)H2PO4	7.3	+	5	Glutathion Allopurinol	PEG 35kDa (1g/L)

Lifor®	16	98	Phosphates	7.07	+	?	?	?

Polysol®	15	120	(K)H2PO4 HEPES Histidine	7.4	+	5	Glutathione Acid ascorbic	PEG 35kDa (20g/L)

SCOT 15®	5	118	HCO3-	7.4	+	0	-	PEG 20 kDa (15g/L)

**Flush solutions**								

Carolina RS®	5	115	(K)H2PO4	6,5	+	1	Glutathion Allopurinol	HES (50 g/L)

**Dynamic preservation solution (for hypothermic perfusion machine)**								

KPS-1®	25	97.5	(K)H2PO4 HEPES	7.4	+	5	Glutathion	HES (50 g/L)

PERF-GEN®	25	100	(K)H2PO4 HEPES	7.4	+	5	Glutathion	HES (50 g/L)

MPS®	25	100	(K)H2PO4 HEPES	7.4	+	5	Glutathion	HES (50 g/L)

**Kidney hypothermic perfusion machines**								

**Machine**	**Solution type**		**Pulsatile perfusion**		**Temperature**		**Oxygen supply (100**%**)**	

LifePort®	KPS-1® MPS®		-		4°C		No	

WAVES®	PERF-GEN®		+		4°C		100%	

Kidney Assist-Transport®	KPS-1® MPS®		-		4°C		100%	

HTK (Custodiol®, Dr Franz Köhler Chemie GMBH, Alsbach-Hähnlein, Germany); UW (University of Wisconsin, Alumni Research Foundation, Madison, WI, USA); Celsior® (Genzyme Corporation, Cambridge, MA, USA); IGL-1® (Institut Georges Lopez, Civrieux d'Azergues, France); Lifor™ (Lifeblood Medical, Freehold, NJ, USA); Polysol® (Doorzand Medical Innovations B.V., Amsterdam, The Netherlands); SCOT15® (MacoPharma, Tourcoing, France); Carolina RS® (Carolina Rinse Solution, University of North Carolina, Chapel, USA); KPS-1® (Organ Recovery Sytems, Chicago, USA and Brussels, Belgium); MPS® (Belzer MPS® UW Machine Perfusion Solution, Bridge to life; Columbia, USA); PERF-GEN® (Institut Georges Lopez, Civrieux d'Azergues, France); LifePort® (Organ Recovery Sytems, Chicago, USA and Brussels, Belgium); WAVES® (Institut Georges Lopez, Civrieux d'Azergues, France); Kidney Assist-Transport® (Organ Assist B.V, Groningen, The Netherlands); HEPES (4-(2-hydroxyethyl)-1-piperazineethanesulfonic acid); HES (hydroxyethyl starch); PEG (Polyethyleneglycol).
